# Typhoid Fever in North Western Virginia

**Published:** 1856-10

**Authors:** James E. Reeves

**Affiliations:** Phillippi, Barbour Co., Va.


// BUFFALO MEDICAL JOURNAL

AND

MONTHLY REVIEW.

VOL. 12. OCTOBER, 1856. NO. 5.

### ORIGINAL COMMUNICATIONS.

ART. I.— *Typhoid Fever in North Western Virginia.* By JAMES E. REEVES, M. D., Phillippi, Barbour Co., Va.

DR. HUNT: As typhoid fever has become very common in this part of Virginia, I have concluded to present to the readers of your Journal, an account of the disease as it occurs in this region, deduced from an analysis of 110 consecutive cases in my practice during the five years ending January, 1856. In presenting the following communication, I offer as an apology the importance of country reports of any given disease, when truthfully made, in order that their results may be compared with city and hospital records, thereby affording the profession valuable data for determining its correct nature and treatment.

Typhoid fever, in this region of country, has completely supplanted the bilious remittent form, of by-gone years. So frequently has typhoid fever scourged this part of Virginia during the past ten or twelve years, that even now, when it is much better understood, the *name* in some families is but the recollection of the days of death and mourning.

I propose a division of the symptomatology of typhoid fever, as regards the gravity of the disease, into three distinct forms:

1*st.* *The Simple, or Mild.*

2*d.* " *Intermediate.*

3*d.* " *Malignant,* including The Fatal.

This arrangement, I think, will be found preferable to any which our authorities supply. The duration of each of these forms is various. There is no other acute disease in which the period of convalescence is more uncertain. The duration of the *mild form* may not exceed nine days, or it may be protracted to twenty-five days; in this event, neither the patient nor his physician observing any material change after the first ten or twelve days, except that there has been a gradual decrease of bodily strength: or the case thus mild for several days, may pass into the *intermediate form,* and yet not run a longer course, before the more aggravated symptoms pass off: or after having completed a longer or shorter measure of the symptoms of this form, may still take on the severer class of symptoms belonging to the *malignant,* and yet the duration of this case may not exceed the limits of one of the *mild form.* But it is proper to remark, that the *malignant* form of typhoid fever, as I shall speak of it, is not always the consequence of the existence first, of the milder forms. In some instances, the disease is essentially malignant in the beginning; and in these, also, the duration is alike various. But these differences of duration will be alluded to again; I enter now upon a description of the symptoms of typhoid fever, and First, The Prodromata.

The accession of this disease is quite different in different cases; some come on suddenly, without any premonitions. In others the period of incubation is extended from two to six days: the patient complaining the meanwhile of sensations of mental and bodily languor; inability to perform his accustomed exercise; a dull, heavy pain in the head, back, and limbs, with a degree of bodily soreness almost amounting to positive pain. At the same time he experiences feelings of chilliness alternating with heat; appetite greatly impaired, with occasional nausea, some unnatural thirst, with a dry insipid state of the mouth; the pulse grows quick, usually 90 to 95 per minute; the countenance expresses indifference; his sleep is interrupted on account of an evening exacerbation of febrile excitement, and the morning arrives, bringing with it no marked alleviation of the symptoms. In some instances, however, there seems to be a morning remission of all the troublesome feelings, and sometimes free sweating occurs. Again, I have seen this relaxation of symptoms occur only on alternate mornings, which may continue for several days after the disease is fully formed, though not so distinctly marked as during the formative stage. There may be at the same time a moderate looseness, or a costive state of the bowels, with now and then a few wandering pains in this region, with a sense of fullness of the stomach: the latter attracting the particular notice of the patient, and he wonders why it is so, adding that "for two or three days I have not been able to eat anything." Hour after hour these ill-feelings grow more distressing, when at last a sensation of chilliness more severe and prolonged is experienced, with an aggravation of the headache, backache, &c. The patient is now gladly induced to take his bed, although a few hours before he manfully protested against the idea of a *thirty days' bed*, flattering himself that his feelings of *mal aise* were only those resulting from the effects of a severe cold, too trifling *to demand medical aid*, and that they would pass off of themselves.

Such is the outline of the formative stage of typhoid fever in the maximum number of cases. In the minimum number, this regular succession of prodromata is not observed: headache, backache, chills, nausea, and vomiting, with a rapid prostration of physical strength, seem to be the *instant* accompaniments of this mode of attack.

The mode of access in the 110 cases alluded to, in which I endeavored to ascertain this point, was as follows:

*Males.*

| | |
|---|---|
| Access sudden in - - - - | 16 cases. |
| 2 days formative stage in - - - | 7 " |
| 3 " " " " - - - | 21 " |
| 4 " " " " - - - | 9 " |
| 5 " " " " - - - | 3 " |
| 6 " " " " - - - | 1 " |
| | 57 cases. |

*Females.*

| | |
|---|---|
| Access sudden in - - - - | 20 cases. |
| 2 days formative stage in - - - | 11 " |
| 3 " " " " - - - | 15 " |
| 4 " " " " - - - | 5 " |
| 5 " " " " - - - | 2 " |
| 6 " " " " - - - | 0 " |
| | 53 cases. |

It will be perceived that in those cases in which the access was sudden, and those in which the formative stage was lengthened to two days, a slight preponderance is exhibited in favor of females; but in those which it was protracted to three, four, five, or six days, form a majority in favor of males.

The symptoms peculiar to the *simple or mild form of typhoid fever* in these, were as follows: A marked increase of severity of the former symptoms; the pulse has become more frequent—say 95 to 100; increased heat of skin; urgent thirst, the patient calling for water every ten or fifteen minutes; violent headache, and throbbing of the temples; face flushed and apparently swollen; tongue covered with a white or yellowish fur, occasionally dotted with an *apthous* exudation; urine diminished in quantity and highly colored; wandering pains in the stomach and bowels; nausea, and occasionally free vomiting; entire disgust for food; bowels inclined to looseness, slightly tympanitic, and a gurgling noise and tenderness on pressure over the coecal region. *This gurgling noise I have found to be a constant accompaniment of typhoid fever.* The patient passes restless nights; his sleep is disturbed, and even rendered painful by frightful dreams—frequently crying alond, as if really an actor in some horrible tragedy. During his dozing moments through the day, painful impressions trouble his mind. A common impression is, that his body is severed in pieces, one portion at one side of the bed, another at the other, which he anxiously attempts to reunite, and at the point when he is about to succeed in his imaginary undertaking, some other accident befalls him, when he suddenly awakes to communicate the torture of mind he has undergone.

Toward the fifth day the headache generally passes off, but in its stead increased obtuseness or drowsiness of manner is observed. During the progress of an ordinary case, the bowels are moved two or three times a day, the stools in appearance and consistence "resembling new cider;" there is a daily exacerbation of fever, most generally toward evening, sometimes twice a day, during which, red spots about the size of a half dollar occasionally appear, first upon one cheek and then on the other, rarely upon both at the same time; or in the absence of the spot on the cheek, the *nose* may become red and shining, giving to the patient rather an unusual appearance; there may be also occasional epistaxis, though seldom profuse; the tongue, after the first four or five days, begins to look red around the tip and edges, sometimes it will part with its fur first in patches—one side clean and of a bright red—while the other half is covered with a thin white fur; it may be moist, or inclined to dryness, and somewhat trembling when protruded. During the entire advance of the case, connected with the above symptoms, will be observed more or less cough, usually slight, and toward the close of the disease tolerably free expectoration. But among the number of symptoms enumerated, no one is so marked and constant as debility; and the degree in which it exists at any period, cannot be correctly estimated by the patient unless when he attempts to set up out of bed: *a soft couch and cool water* he would not exchange for any other luxury.

But to complete the description of this form of the disease, I mention another accompaniment: the *rose-colored* spots, the regular appearance of which I have seldom failed to observe at some period of the disease. I cannot say this much of the transparent vesicular eruption called *sudamina*, never having noticed it but in a few cases, all of which occurred in the severer form designated as the intermediate.

I have thus attempted to describe the symptoms, with their modifications, peculiar to the *mild* form of typhoid fever, all, or a majority of which, enter into the composition of every case of the disease.

The duration of this form, like each of the others, is quite uncertain; yet the amelioration or aggravation of certain symptoms at certain times, either point to approaching convalescence or portend the conversion of this form into one of greater severity. When the case is about to terminate in convalescence from this form, the febrile exacerbations become less marked, and of shorter duration; the pulse becomes soft and less frequent; the tongue loses its coating; the discharges from the bowels become more consistent; thirst diminished; the features shrink, the countenance brightens, and the appetite returns.

Of these 110 cases 57 were of the *mild* form of the disease, the duration of which was as follows:

*Males.*

| | | | |
|---|---|---|---|
| In 1 case the duration was | - - - | 9 days. |
| " 2 " " " | - - - - | 10 " |
| " 1 " " " | - - - | 12 " |
| " 3 " " " | - - - - | 13 " |
| " 6 " " " | - - - | 14 " |
| " 5 " " " | - - - - | 15 " |
| " 1 " " " | - - - | 15 " |
| " 6 " " " | - - - - | 17 " |
| " 4 " " " | - - - | 18 " |
| 29 | | |

*Females.*

| | | | | |
|---|---|---|---|---|
| In 1 case the duration was | - | - | - | 9 days. |
| " 1 " " " | - | - | - | 10 " |
| " 3 " " " | - | - | - | 12 " |
| " 1 " " " | - | - | - | 13 " |
| " 4 " " " | - | - | - | 14 " |
| " 9 " " " | - | - | - | 15 " |
| " 5 " " " | - | - | - | 17 " |
| " 2 " " " | - | - | - | 18 " |
| " 1 " " " | - | - | - | 23 " |
| " 1 " " " | - | - | - | 25 " |
| 28 | | | | |

From the above it will be seen that a majority of the cases of this form terminated between the thirteenth and eighteenth days, reckoning from the time the patients first took to their beds. But it should be remembered, however, that the date at which the patient takes to his bed will not always be the precise date of the full development of the disease, but it is generally the case. I have seen one or two patients suffering from the mild form of the disease, who did not take to their beds for more than an hour or two at a time during the day, throughout the entire course of the disease, but wandered about the streets the object of remark.

But instead of the amelioration of symptoms above mentioned, as marking convalescence from the *mild form,* the case becomes exasperated; we have another distinct and more formidable phase of the disease to contend with.

*The Intermediate Form.* The most prominent changes that are to be observed in this form are these:

The febrile exacerbations become more prolonged, and the remissions subsiding less frequently with perspiration; the pulse becomes gradually weaker, and more frequent, say 110, 15, 20, or 30 to the minute; the tongue previously moist, now inclines to dryness, and takes on a thicker coating. As the case advances the condition of the tongue becomes peculiar, its edges are thin and red, and apparently contracted, its surface concave, taking more the form of the bowl of a spoon with its point somewhat rounded and slightly cracked, than of a tongue. Frequently there will be a dark brown stripe, from a half to three-fourths of an inch in width, running down the middle of the tongue as far back as can be seen, and when protruded it trembles, telling us how much strength *yet* remains. There is also, at the same time, an accumulation of *sordes* on the lips, gums and teeth; twitching of the tendons; increased somnolency; duskiness of the countenance; and greater confusion of mind. The patient does not call so often for cool drinks: he seems insensible, in a great measure, to his wants. If there is a desire to go to stool, he does not make it known to his attendants until just at the minute he feels that the discharge must take place, and he will suffer his bladder to become enormously distended, sometimes, without an effort, or even a desire, to empty it. Partial deafness is quite a common attendant upon this form of this disease. In one instance, my patient being a female, I had to speak at the top of my voice to be heard. Wandering of the mind, more or less, is most always present. At first the mind of the patient wanders in the night only, but as the case advances, this confusion occurs during the day, and sometimes very strange impressions trouble the mind. It is quite a common thing for patients suffering from this form of the disease, to imagine the foot posts of the bed converted into uncomely personages, placed there to make all sorts of grimaces, and they will frequently start up in anger to drive them from the room, the effect of which dispels the illusion, and they return to their quiet pillow wondering at themselves.

Coincident with the above, there are other and significant symptoms having peculiar reference to the state of the bowels. Upon an examination of the abdomen, a marked increase of tympanitic distension will be observed. Diarrhœa is more or less present, always proportioning the amount of meteorism to the frequency of the discharges. It is sometimes slight, causing not more than two or three stools in a day, and at others, as many as ten or twelve in as many hours. The dejections, for the most part, are darker and more offensive than in the *mild* form of the disease, and are frequently attended with very acute pain, most generally referred to the umbilicus.

The urine is deeply colored, voided in less quantities, and occasionally with much difficulty. When there is a disposition on the part of the patient to empty the bladder, he cannot always succeed in his effort, and unless relieved by the catheter much suffering is the consequence. It is in the region of the bowels that the *rose-colored* spots generally first make their appearance. Sometimes they are but few in number, and at others spreading over the entire breast and shoulders. I have found this eruption to a greater or less degree in every case where I have searched for it.

*Sudamina*, as before said, I have not so often observed; but whenever found was always in this *form* of the disease preceded for a day or two at intervals by free perspiration.

Epistaxis, when it occurred, was always mose profuse than in the milder form. In two cases the loss of blood in this wise was quite unusual, being not less than twelve to twenty-four ounces. Both of these patients recovered.

Now, this form of typhoid fever, as above described, may go on undetermined for a short or longer time, flattering us at one time with an approach of a speedy convalescence, at another, exacting our fears. It is not at all uncommon for us to see the tongue clean off, become moist, the countenance brighten, diarrhœa and tympanitis diminish, and the skin become moist, when without the slightest apparent cause, there is a sudden aggravation of all the symptoms: the tongue, just rid of its coating, becomes dry, red, cracked and bleeding; fresh sordes accumulate on the lips and teeth; frequent desire to stool: at one time a free passage, dark and offensive, at another small, with a corresponding increase of tympanitis and abdominal tenderness; pulse more frequent, and less resisting; previous duskiness of the countenance, and wandering of the mind, twitching of the tendons, skin hot and dry. This mutation of symptoms may last but a day or two, and the patient recover without necessarily taking on the graver symptoms reserved as peculiar to the malignant form. When the case is to convalesce from this form, this aggravation of symptoms just named, as rapidly subsides: the tongue coats over again with a thin white fur, and the state of the pulse, the bowels, and the mind, become as favorable as before; and the symptoms continue to improve, until convalescence is completed.

The duration of the *Intermediate* form, like its kindred, is not always the same. But to be understood: I said in an early part of this paper, that a case of this form—the intermediate—may not run a longer course than one in which the disease was mild throughout, (*i. e.*) a case mild in its character at first, may, after the lapse of several days, become aggravated, and present the severer class of symptoms just described, and end in convalescence as soon as one that did not partake of this increase of symptoms. Therefore, in speaking of the duration of the *Intermediate* form, it will be remembered that I include the whole time, from the date of the first development of the fever to the establishment of convalescence. I regret now that I did not note the precise dates when the mild was converted into the intermediate, and the intermediate into the malignant form. If this had been done I could have appropriated to each phase of the disease its exact duration.

Reckoning, therefore, as above proposed, the duration of twenty-five cases passing into this form was as follows:

*Males.*

| | | | | |
|---|---|---|---|---|
| Duration of | 2 | cases was | - - - - | 16 days. |
| " | 3 | " " | - - - - - | 18 " |
| " | 3 | " " | - - - - | 20 " |
| " | 3 | " " | - - - - - | 23 " |
| " | 2 | " " | - - - - | 25 " |
| " | 0 | " " | - - - - - | 0 " |
| " | 0 | " " | - - - - - | 0 " |
| | 13 | | | |

*Females.*

| | | | | |
|---|---|---|---|---|
| Duration of | 1 | case was | - - - - | 17 days. |
| " | 4 | " " | - - - - | 18 " |
| " | 2 | " " | - - - - | 20 " |
| " | 1 | " " | - - - - | 23 " |
| " | 1 | " " | - - - - | 25 " |
| " | 2 | " " | - - - - - | 28 " |
| " | 1 | " " | - - - - - | 35 " |
| | 12 | | | |

I now proceed to complete the description of the symptomatology of typhoid fever:

*The Malignant form.* I have already said that typhoid fever may be stamped with malignancy from the beginning, without having necessarily to pass through the milder gradations. This is not generally the case. The majority of cases of this form is preceded, for a longer or shorter period, by each of the simpler forms. To describe the symptoms of this form I shall go back to the point from which I left off, as completing the symptoms of the intermediate form, and endeavor to present a continuous picture of the disease, from the mildest to the most severe form, and then proceed to notice the rapid succession of symptoms that occasionally occur, marking the malignant form in the beginning.

As already stated, it is not uncommon, after a seeming amelioration of all the symptoms, for sudden and alarming changes to occur, exposing the patient to the dangers of a still severer form, the most remarkable symptom of which was, the drying of the tongue after having become clean and moist. When the disease is to be protracted into a graver form, it is generally at this juncture that it becomes apparent; and the case thus aggravated, rapidly proceeds to a final termination, either in recovery or death. Prominent, then, amid this increase of number and severity of symptoms making up the *malignant* form, will be a greater prostration of strength. The patient lies constantly upon his back, and inclines to slip down toward the foot of the bed; his deglutition may be interfered with; the voice becomes enfeebled, unable sometimes to speak above a whisper,—at others it may be much stronger, but he cannot articulate sufficiently to be understood. The tongue takes on a dark and thicker coating, becomes cracked and bleeding. If the patient is asked to protrude his tongue, he will, perhaps, do so, but the movement is slow, and he will likely leave it exposed, and trembling, if not told to take it in. At the same time fresh sordes will be found to collect upon the lips, gums and teeth. The pulse becomes more frequent and feeble. In some instances it is almost impossible to count it for the constant twitching of the tendons of the wrists—*subsultus tendinum.* A general tremor of the whole body is frequently observed, and in such cases, even the bedstead will communicate a like sensation to the body of one who leans thereon. Cough is more or less present—and this should be attentively watched; the breathing is more hurried than before, and occasionally irregular. The heat of the surface may be uniform, or it may be unequally distributed—one part hot while another is cool, or the face and hands may sweat freely, while the rest of the body is dry and parched. If at the same time attention is paid to the state of the bowels, a proportionate degree of disorganization will be found to have taken place there: the tympanitic distention has enormously increased, as to present sometimes "a convex outline from the ensiform cartilage to the pubis," and if pressure is made, the entire abdominal region appears thin, hard and resisting, "as though its walls were made of pasteboard." The stools are more or less frequent, very offensive, and in color and consistence may resemble brown paint, or even *tar;* and the coating these discharges give to the pot is not always easily removed. The urinary discharge has also undergone a commensurate degree of alteration. While the quantity of urine voided at a time has become less, its color is that of dark ley, and not unfrequently little particles of coagulated blood are deposited at the bottom; or it may be retained, requiring the repeated use of the catheter. It is in this form of the disease *always* that we observe the most frequent and profuse hæmorrhages to occur. Sometimes the urine is retained and dammed up by firm coagula in the bladder. In one case I

had to break up and dilute this coagulum by injections into the bladder of flax seed tea, before the urine could pass out through the catheter; and yet notwithstanding this operation had to be performed regularly for several days, the patient recovered. Indeed I have seen free hæmorrhages take place from the nose, gums and bowels, following each other in regular succession in the same case. But the disease has not necessarily to pass through all, or either of these to arrive at a fatal termination: death may take place without them. Neither does the existence of any one or all of these occurrences insure a fatal termination. They stand as *probabilities* of a fatal termination, but I have seen as many cases recover as die, in which they were present.

Delirium is a common attendant upon this form of the disease. It may be attended with wild and violent agitation, but more frequently is of that species called *low* and *muttering;* the patient picks at the bed clothse, or at imaginary objects in the air; his stools become involuntary, and a sickening odor exhales from his body. Along with these the vitality of the skin becomes so feeble, that gangrenous eschars form on the hips, sacrum and shoulders.

Finally, if after a long and tedious struggle between the efforts of nature to cure, and the powers of disease to kill, nature is about to triumph, the pulse grows less frequent and acquires strength; if there has been delirium or stupor, it gradually subsides; *subsultus tendinum* ceases; the tongue becomes moist and soon parts with its coating; the discharges from the bowels are of a lighter color, and more consistent, and less offensive; there is a more abundant secretion of urine; the surface becomes moist and of uniform temperature; the features shrink; and the appetite returns. But if disease gain the mastery, it may accomplish its purpose of death in different ways. If it is to be by *coma*, the low muttering delirium from which the patient could at first be aroused, gradually becomes more profound; the eye balls are upturned, and roll from side to side; the pulse beats more rapidly, and with gradually decreasing regularity and strength; the respiration grows quicker, and automatic efforts are made to expel the mucus which chokes up the lungs, and begins to rattle in the throat; a cold, clammy perspiration breaks out upon the skin; the extremities become cold, this coldness gradually extending toward the trunk, when at last there is a final struggle, and death reigns.

Another mode of death we sometimes see, is by *asthenia.* Death occurring in this manner does not take place early. I have never seen it to occur until after the third week, and always preceded by large losses of blood by hæmorrhage, after which, swelling and ulceration of the parotids, petechia, purple spots, and the appearance of large *bullæ*, containing a mixture of blood and water, which soon become *pustular*, upon various parts of the body, closed the scene. Yet, notwithstanding this abundant measure of putrescency, I have seen the mind remain unclouded to the very last hour. When perforation of the bowels occurs, there is a sudden supervention of very acute pain in the abdomen — at first circumscribed, but soon spreading over the entire belly. (See fatal case female, age 48.)

Such, now, are the symptoms of a case of typhoid fever, either mild throughout, or after having been mild for a longer or shorter time, passing into the *intermediate* form, and convalescing from this, or entering into one of still greater severity — the *malignant* form.

I will now briefly speak of those cases, occasionally occurring, in which a degree of malignancy is observed in the beginning, or very soon thereafter. Of twenty-five cases of the malignant form, nine presented symptoms of this cast from the beginning: five males and four females. In each of these cases there were strong manifestations of cerebral disorder in the beginning: aversion to light and sound; watchfulness; confusion of mind, and delirium — the latter occurring during the first two or three days of the disease. During the further advance of these cases, the delirium became continuous, and occasionally violent; the eyes were injected, the countenance of a dusky hue; startings of the tendons; skin intensely hot; bowels a little inclined to be sluggish; the tongue thickly coated, of a dark color, and inclined to dryness; pulse not very firm, but frequent, amounting to 115 or 120; bleeding at the nose, which was sometimes free; the urine diminished, and highly colored. Such are the symptoms usually attending a case thus suddenly attacked, for the first seven or eight days, after which, instead of the previous costiveness of the bowels, a diarrhœa frequently sets in, with a degree of tympanitis not before observed, and a sudden prostration of strength. To this condition is added a pulse less characterized by strength; heavier *sordes* accumulate on the lips and teeth; the tongue is very dry, and its coating looks as though it were made of black varnish — for it glistens — and when protruded, trembles very much. The case thus far described, may go on undetermined for several days longer, or even for several weeks. When it is to result fatally, the case takes on early the additional symptoms belonging to death by coma, as already enumerated.

The duration of the nine cases alluded to was as follows: In 2 cases, the duration was 12 days; in 2 cases, 15 days; in 2 cases, 18 days; in 1 case, 21 days; in 1 case, 25 days; in 1 case, 60 days.

Among these were two fatal cases, male and female, age of the former 56 years, of the latter 21 years; the duration of the disease in the male twenty-one days, in the female eighteen days.

Of the 19 remaining cases belonging to the *malignant* form, the duration was as follows: In 1 case, the duration was 12 days; in 2 cases, 15 days; in 4 cases, 18 days; in 1 case, 21 days; in 1 case, 23 days; in 2 cases, 26 days; in 5 cases, 28 days; in 1 case 30 days; in 2 cases, 31 days. Males, 10; females, 9.

Of the 7 fatal cases occurring among these the duration was:

28 days in 4 cases: 3 males and 1 female; ages, 11, 18, 23, and 48 female.

26 days in 2 cases: males—ages, 19 and 24.

23 days in 1 case: female—age, 19.

Thus, making in all, 9 fatal cases.

### *General Remarks.*

From the foregoing detail, it will be seen that amid the multitude of symptoms belonging to typhoid fever, those relating to sensorial disorder, stand among the most prominent; that these symptoms vary in degree of severity from mental languor or inability in the beginning to the wildest delirium; and that the subsidence or diminution of these constitute one of the earliest and surest signs of approaching convalescence.

It is worthy of remark, however, that while it is usual for the mind to have regained its entire soundness by the time convalescence is fairly begun, that occasional cases occur in which a morbid condition of the mind remains even after the patient is able to be out of doors. A case of this kind is included among the nine cases spoken of, presenting a severe form of the disease in the beginning. The case was that of a young man, aged 18, of robust form; business that of a clerk in a store, and of an active turn of mind, who, after complaining of the premonitory symptoms of the disease for a day or two, was taken down in the manner above described. Delirium came on early; and after the first four or five days, was attended with wild and violent agitation, to such a degree as to require the constant attention of his friends to keep him in bed. At the end of twenty-one days, however, the delirium had entirely subsided, and he began to relish a little food; and by the thirty-second day he could be out of doors. From the time the delirium passed off up to this last named date, he had not exhibited the slightest aberration of mind; but, very much to the surprise of his friends, on the next day—the thirty-third—he accused the girl of the house of having stolen his account book, "with a leather back," in which, he said—he had charged several of his friends with large sums of money loaned: money that he had drawn at lottery, amounting in all to $10,000. His mother, who was present at the time, endeavored to convince him that he was wholly mistaken, that he had certainly not been so fortunate as to gain money in this wise, and that he was troubling himself about a matter altogether imaginary. At this he became angry, declaring most solemnly that his whole statement was true; that he had had the money in his own hands, charged the amounts loaned in the aforesaid book—that he was not mistaken; and accused her of having joined in with other of his debtors to defraud him of the whole sum. In a few days, however, he became convinced of his error of mind, and could laugh over it heartily.

Typhoid fever is occasionally complicated with other diseases. I have seen two cases in which it was complicated with pneumonia in the beginning. One case recovered.

In the one fatal case—of twenty-three days duration—a female, erysipelas attacked the ear on the evening of the twenty-first day of the disease, which spread rapidly over the head and neck, killing the patient by the twenty-third day.

Pregnancy and lactation, I have not found to have any material influence upon the disease. In the twelve cases of the intermediate form pregnancy existed in three, the advance of which was two months in two, and three months in the remaining one. Neither of these miscarried. Among the nine cases that passed from this form into the malignant form, pregnancy existed in one case, at an advance of two months. Miscarriage occurred on the tenth day of the disease, and was not followed by any serious result. The patient began to improve by the twenty-first day.

Of those who gave suck, two were of the *mild*, and one of the *malignant* form. The duration of the disease in the former was fifteen days in one, and eighteen days in the other. In the latter, the duration of the disease was thirty days. In neither of the former was the child denied its usual amount at the breast. In the latter case, of the malignant form, during the second week, the secretion of milk was entirely suspended; the child was, in consequence of it, removed to a neighboring family, and not permitted its mother's breast for two months, when an abundant secretion again took place.

Taking into account physiological differences, I have not seen the course of the disease in infancy and childhood, materially different from that in adults. In two cases the disease appeared as early as the eighteenth month, (the mothers suffering from the disease at the same time); and from the third year up to the twelfth, there were nineteen cases. Including the two at eighteen months, fourteen were of the mild form, four of the *intermediate*, and three of the malignant. The duration of the disease varied between nine and twenty-eight days. In the case of the last named duration (age 11) profuse hæmorrhage from the bowels took place on the twenty-third day, followed by petechia, &c., terminating the life of the boy on the twenty-eighth.

### *History and Causes.*

Typhoid fever was not recognized in this country prior to the year 1837. In October of that year, a gentleman from this neighborhood, who had been on a visit to Athens Co., Ohio, was brought home sick of the disease, called at that time in Ohio, "*slow fever.*" In three or four weeks after his arrival, his mother, father, and three brothers, were taken down with the disease; the mother died. The last of November following, six of another family who was near by, took the disease; one died. In December it appeared in a third family, five of these had the disease, and three died. From these it sprang up in another family, five took the disease, and one died. The last of January, 1838, nine of another family had the disease, and four died. During the following spring and summer, two other families took it, nine of these died. In the winter of 1838 and '39, several other families took the disease, amounting in all to twenty cases; of these six died. From this up to the winter of 1840 and '41, there were occasional cases springing up throughout the country. But during the fall and winter of 1841, the disease appeared in another neighborhood, ten miles distant; several families were attacked, among which there occurred ten cases; four died. In the fall of 1842, a merchant returned from the north, sick with the disease (his neighborhood alike previously free from the disease); several of his family took it, but none died. The following winter it appeared in another neighberhood, six had the disease, and three died. From this date up to the winter of 1843, there were but few cases occurring; but during the next spring, it made its appearance in still another neighborhood, some ten cases occurred, and four died. During the year 1845, it was generally prevalent in the country; about thirty cases occurred during this year. From the close of the year 1845, up to the year 1851, the disease became less common; but during the spring and summer of this last year, the extent of its visitation exceeded that of any former year; and the citizens of our village, particularly, suffered severely from the disease.*

Philippi is situated about ten miles west of the Laurel Mountain, on the Tygarts Valley River, forty miles from its junction with the Monongahela, which for two miles above, and five miles below us, has a sluggish current. Across the river, immediately opposite Philippi, is a small village, George Town. The bank on that side is very low for a few hundred yards, spreading out into a level in extent of about ten acres, and bordered on both sides by high hills. The greater portion of this ground is inundated at every considerable rise in the river. In the spring of 1851, the construction of a bridge connecting Philippi with George Town, was commenced; adding about thirty to the population of this last named village. Among these workmen whose business it was to stand in the water, typhoid fever began; from these, it occurred among the workmen in stone upon the banks; and from these to the citizens generally. Our side of the river, Philippi, was next attacked, extending from family to family. Several of the workmen at the bridge, on being taken sick, went to their houses in various parts of the country, forming starting points of the disease there. Other hands were employed to fill the places of those taken sick, but were soon likewise taken down. Fresh workmen were again and again secured; but the disease as successively continued to attack them, until at last, during the year 1852, the work was, in consequence of it, almost completely suspended.

In September of this year, there was a menagerie at Philippi, largely attended by the people of the county; the following night nine persons in attendance during the day, were taken sick with the disease.

From 1851 to 1856, our country has never been entirely free from the disease for more than a month or so at a time. During this period the disease has been wandering about the country, at one time mild, at another very severe, attacking one neighborhood this year, another the next; and then, perhaps, returning to the locality it scourged the year before. The last fall and winter the disease was uniformly severe. The summer was wet throughout; vegetation grew rapidly, and was abundant. Heavy frosts appeared early, and the offensive exhalation from the decomposition of vegetable matter was remarkable. Hæmorrhage from the bowels was of frequent occurrence at this season.

---

* I am wholly indebted to my friend, Dr. E. D. Talbott, of Philippi, for the above early history of typhoid fever in this country.

Within the limits of Barbour county, during the past six years, according to my own observation, and the observation of my medical brethren of the county, there have been not less than 600 cases of typhoid fever. Of these about sixty have died. In Philippi the disease has nearly gone through with the entire population above twelve years of age.

*Contagion.* What other evidence besides that unavoidably adduced in the foregoing history of the disease in this county, have I to add to prove the contagiousness of typhoid fever? I could bring forward abundant testimony, but I deem this unnecessary; and I shall content myself with the detail of a single instance:

Riley M——, aged 21, a farmer, visited an acquaintance sick of the disease, Oct., 1853, distance some ten miles. There was no fever in his neighborhood at the time; but in two weeks after his visit to this patient, he took the disease at his father's dwelling, and died. In ten or twelve days after his death, his sister, two brothers, mother and father, took it; the mother died. Among those who visited Mr. M., during his illness, was a cousin, a girl of nineteen, and a male friend, both of whom immediately contracted the disease, and the cousin died. From these the disease spread throughout the neighborhood.

The above is but the history of many such cases. The contagious feature of typhoid fever, together with another—the perfect immunity to subsequent attacks,—is well understood by the people at large throughout this country.

*Age* has some influence on the permission of the disease. Of the 110 cases, upon which this paper is founded, the several ages were as follows:

*Males.*

| | | | | | |
|---|---|---|---|---|---|
| 1 at 18 months. | | | | | |
| 6 | from | 3 | to | 6 | years. |
| 2 | " | 6 | " | 9 | " |
| 3 | " | 9 | " | 12 | " |
| 3 | " | 12 | " | 15 | " |
| 6 | " | 15 | " | 18 | " |
| 3 | " | 18 | " | 21 | " |
| 13 | " | 21 | " | 25 | " |
| 10 | " | 25 | " | 30 | " |
| 3 | " | 30 | " | 35 | " |
| 2 | " | 35 | " | 40 | " |

| | | | | | |
|---|---|---|---|---|---|
| 3 | - | from | - 40 | to | 50 years. |
| 2 | - - | " | - - 50 | " | 60 " |
| 57 | | | | | |

*Females.*

| | | | | | |
|---|---|---|---|---|---|
| 1 at 18 months. | | | | | |
| 2 | - | from | - 3 | to | 6 years. |
| 4 | - - | " | - - 6 | " | 9 " |
| 2 | - | " | - 9 | " | 12 " |
| 9 | - - | " | - - 12 | " | 15 " |
| 4 | - | " | - 15 | " | 18 " |
| 7 | - - | " | - - 18 | " | 21 " |
| 9 | - | " | - 21 | " | 25 " |
| 7 | - - | " | - - 25 | " | 30 " |
| 2 | - | " | - 30 | " | 35 " |
| 1 | - - | " | - - 35 | " | 40 " |
| 3 | - | " | - 40 | " | 50 " |
| 1 | - - | " | - - 50 | " | 60 " |
| 1 | - | " | - 60 | " | 65 " |
| 53 | | | | | |

*Season.* The autumnal and winter months seem most productive of the disease. The 110 cases occurred as follows:

January, 12; February, 5; March, 7; April, 5; May, 4; June, 9; July, 3; August, 8; September, 9; October, 16; November, 22; December, 10.

*An Outline of the Treatment.*

The treatment of typhoid fever, in this region, has been considerably modified during the last several years. The old aphorism, *aux grands maux les grands remédes*, has proven itself to be wholly unsuited to the successful management of this disease; and we have learned to substitute for it, with much better encouragement, a reliance to a considerable degree upon the *vis medicatrix naturæ.*

As it was seen that typhoid fever presents different grades of severity, so will it appear that the treatment should be alike varied to meet the indications of each form. I shall therefore speak of the treatment in the same order that I have classified the symptoms.

*The Mild Form.* General bleeding I have very rarely practiced in either form: but three times, I believe, in the 110 cases, all of which were among those of unusual severity in the beginning. But the local abstraction of blood, by cups to the *nucha* and temples, when headache is severe in the beginning, and subsequently over the bowels when tenderness exists, I resort to freely. An emeto-cathartic of calomel and ipecac, in the beginning, I am much in favor of, and never omit it unless the bowels are too much purged without medicine. After this I am in the habit of being content with a daily change of the body and bed linen; sponging the surface, when hot and dry, with cool water; cups, and mush poultices to the bowels, when tenderness exists; a draught of effervescing water, composed of citric acid and bicarbonate of potassa, several times during the day; a Dover's powder at night to procure rest, followed by one in the morning, if the bowels are discharging too frequently; a little spts. nitre dulc., after the first few days; a cloth wet in cold water and kept to the head, when headache is present; cold water for drinks, but not in too great quantities at a time; and the regular allowance of a little food.

*The Intermediate Form.* In this form, instead of sponging the surface with cold water, tepid water with vinegar I think is best. Instead of the effervescing water, I substitute the spiritus mindereri in the dose of a half fluid ounce every three or four hours. When there is tenderness of the bowels, I employ free cupping—not with small glasses, but the largest ones to be had—followed by mush poultices as warm as can be borne, with now and then a mustard cataplasm, large enough to cover all over the bowels; these to be used until pain and tenderness are relieved. It is always best that the bowels should be suffered to remain moderately loose. Two or three discharges a day I think are of service, and if these do not take place naturally, some mild laxative should be administered. But should there be too great a looseness of the bowels, which is most commonly the case, they may be restrained by a dose of Dover's powder, three or four times a day. It is in this, and the subsequent form, that the good effects of opium, when administered with due discrimination, are to be witnessed. A favorite formula with me is, tinct. opii, Hoffman's anodyne aa. iiiʒ, spts. lavender comp. iiʒ. Dose, a teaspoonful, given in the morning. For a night dose, six or eight grains of Dover's powder, or opium with camphor, answers an excellent purpose.

In many instances, the above treatment is all that is necessary to conduct the case to an early convalescence. But should it happen, that after the tongue has cleaned and become moist, the diarrhœa and tympanitis diminish, there is a sudden exasperation of these symptoms, the tongue just rid of its coating becomes red, smooth and dry, with an increase of diarrhœa and tympanitis, subsultus tendinum, &c., then opium with camphor should be freely administered. For this peculiar form of typhoid fever, as represented by the state of the tongue, other remedies have been highly extolled. Oil of turpentine and argenti nitras come to us upon good authority. I have used both of them, and sometimes with an apparently good result. I resort to them with good *results*, when opium is not admissible. But of the uniform good effects of opium, when the brain is not involved, for this condition of symptoms just described, when administered liberally, and with the addition of camphor, the success of the cases that have passed through my hands has established a confidence I can repose in no other remedy.

I said, when describing the symptoms of this *form*, that this sudden change of symptoms might occur, and yet the patient enter into convalescence without taking on the malignant form. When this favorable result is about to take place, the tongue, from being red and dry, coats itself with a thin, whitish fur, becomes moist; the pulse grows soft and less frequent. But if the case is to be protracted into

*The Malignant Form*, then our last object is to assist nature in her final struggle, to prevent the termination, by death, in the mode most likely to occur. In those cases with a red and dry tongue, as they pass into this form, hemorrhage from the bowels not unfrequently follows; and in these I continue the opium in as large doses as the patient can well bear, with a proper proportion of the sugar of lead. For hemorrhage from the gums, I have found nothing which affords more prompt relief than a solution of creosote, as prepared by Messrs. G. W. Carpenter & Co., Phila. For epistaxis, when injected into the nostril, the creosote answers equally a good purpose. For the extreme debility attendant upon this form, tonics and stimulants become absolutely essential; but in the allowance of these, the state of the pulse and the skin must be carefully considered. Washing the surface with brandy and water, and the internal administration of carbonate of ammonia, wine, and the sulphate of quinine, are eligible remedies. In a few cases I have found it necessary to resort to pure brandy with quinine, and with happy results. Opium is not the least in importance of these remedies. Whenever the brain is not involved, it is *multum in parvo*. In the management of this form of the disease, my reliance upon opium exceeds that of any other remedy. In connection with camphor, I have seen it quiet delirium, induce moisture of the surface, and promote gentle sleep, from which the patient has awoke quite a different person.* But it very often happens that opium fails to check a wasting diarrhœa. In these cases the bowels need not only an astringent but a tonic. To meet this indication, I have found nothing so well suited as the following injection: "Sulph. quinine, from twenty to sixty grains, dissolved in from two to four ounces of sharp vinegar, and thrown up the bowels two or three times a day." Throughout this form, the state of the bowels, as regards tenderness, should not be lost sight of, but wet and dry cupping, as the case may admit, with the application of mush poultices, and the mustard cataplasms, should be attended to. Blisters over the bowels I have not seen attended with sufficient good to give them importance.

For subsultus tendinum, and a tremulous condition of the body, valerian sometimes acts like a charm. I mean the *powdered valerian*, in doses of a teaspoonful every two or three hours. Retention of urine is a common occurrence. The importance of examining into the state of the bladder daily, has already been sufficiently stated. A tendency to the formation of eschars from pressure should not be forgotten. The parts exposed to pressure should be frequently washed with brandy, and the position of the patient as often changed. When obstinate delirium, or coma, exists, I have seen much advantage result from the timely application of a blister to the nape of the neck, and the administration of a few blue pills. Should the lungs become involved, cupping, wet or dry, and a large blister upon the breast, must be early resorted to. Due attention should be paid to the diet. I have found boiled milk to suit very well.

In those cases that present the symptoms of *a severe form in the beginning*, when the pulse was full and strong, and obvious sanguineous determination to the brain, I have sometimes taken blood from the arm. But in the majority of these cases I have preferred to trust to the effect of the cold douche, and thus far with entire satisfaction. As it is impossible for us to guess correctly the duration of any case of typhoid fever, from any symptoms that may present in the beginning, the general abstraction of blood should be cautiously practiced. But whether we bleed or not, it is important that the bowels should be thoroughly purged. Along with these cases, in the beginning, there is generally a torpid state of the bowels, and requires a larger dose than in the mild form. In all these cases, if the patient

* In the Half Yearly Abstract of the Medical Sciences, No. XVII, is contained a valuable paper on the use of "Opium in Irritable and Anæmic States of the Brain in Fever," by Humphrey Sandwitch, M. D.

be an adult, I give not less than twenty grains of calomel, followed, in due time, if it does not operate, by a dose of sulphate of magnesia, or infus. of senna. During the first few days, free cupping upon the back of the neck, followed by a blister, if delirium is obstinate, cold applications to the head, a blue pill given at night, followed by a seidlitz powder in the morning, is the course of practice I usually adopt, after which I omit the mercury, and pursue the course of treatment before described.

